# Editorial: New omics research challenges for Public and sustainable Health

**DOI:** 10.3389/fmicb.2022.1078865

**Published:** 2022-11-28

**Authors:** Deborah Traversi, Giancarlo Ripabelli

**Affiliations:** ^1^Department of Public Health and Pediatrics, Environmental Hygiene Laboratory, University of the Study of Turin, Turin, Italy; ^2^Department of Medicine and Health Sciences “Vincenzo Tiberio”, University of Molise, Campobasso, Italy

**Keywords:** Public Health genomics, microbiome, epigenetics, prevention, omics science

Genomics play a key role in many pathologic pathways such as in cancer, heart and metabolic diseases (Muin et al., 2010), neurological and cognitive disorders (Migliore and Coppedè, 2022). These different health effects are partly a result of interactions among genes, human behaviors such as diet and physical activity, the environment, and other social factors. The quantitative assessment of gene-environment interactions is a pressing point for the definition of appropriate interventions, both at an individual level and from an epidemiological perspective, and can allow identification of precise preventive strategies (Olden and Wilson, 2000; Torkamani et al., 2018). This is especially true for high incidence diseases such as type 2 diabetes (Sørensen et al., 2022).

The attributable risk for genetic factors is generally low; conversely, the combination of genetic and other risk factors may result in an increased risk, following not only an additive but also synergic and exponential model. The pathway de-codification of such complex outcomes nowadays includes the use of innovative tools developed by omics science, beginning with genetics through to genomics, epigenetics, proteomics, metabolomics, and microbiomics (Gilbert et al., 2018; Knight et al., 2018; Inamura et al., 2022).

Evidence-based data on health risk modulation and the preventive capabilities of microbiome analysis are continuously increasing (Xiao et al., 2022) despite the still uncommon implementation of such analytical tools in health services for the general population (Lewnard and Reingold, 2019).

Nowadays, genetic and genomic screening is limited to persons with a clear high familial risk (Yoshida, 2021). On the other hand, as health benefit strategies, microbiome interventions are limited to the oral consumption of microorganisms and/or substrates (Suez et al., 2019), or fecal microbiota transplantation for the treatment of persistent and antibiotic-resistant *Clostridium difficile* infections (Longo et al., 2015).

Therefore, in this Research Topic, several original articles and reviews have explored how an effective population health gain is possible when omics tools are implemented. In order to guarantee the health service economy, the advantages of omics assays could be indispensable at a population level, without socio-economic selection, and after a preliminary assessment of their medium- and long-term effectiveness.

The contribution “*Evidence of SARS-CoV-2 antibodies and RNA on autopsy cases in the pre-pandemic period in Milan (Italy)*” by Lai et al. is an interesting example of the usefulness of pathogen genetics in outbreak source investigations, particularly when the presence of pathogens in human samples was supported by other molecular evidences. Starting from unusual biological samples, it was possible to trace the origin and diffusion of a pathogen in a specific area.

The article “*System mapping of antimicrobial resistance to combat a rising global health crisis*” by Matthiessen et al. proposed a system mapping tool, which interconnected animals, humans, and the environment in a “One Health” approach. It discussed potentially powerful entry points for system-wide interventions in order to mitigate the spread of antimicrobial resistance, one of the most worrying health issues of the future (World Health Organization, 2022).

The article “*A two-time point analysis of gut microbiota in the general population of Buenos Aires and its variation due to preventive and compulsory social isolation during the COVID-19 pandemic*” by Aguilera et al. presented original data on significant changes in gut microbiota before and after the emergence of the pandemic in a sample of Buenos Aires inhabitants. It discussed how behaviors and confined environments can rapidly affect biological variables related to human health such as the gut microbiota.

The article “*Extremely small and incredibly close: Gut microbes as modulators of inflammation and targets for therapeutic intervention*” by Piazzesi and Putignani discussed molecular mechanisms in the relationship between the gut microbiome and inflammation processes, describing the central role of such interactions in many disease pathways (from chronic gut inflammations to other systemic effects).

The contribution “*Managing the introduction of genomic applications into the National Health Service: a special challenge for health technology assessment in Italy*” by Pitini et al. focused on fundamental aspects of Public Health omics application. It described how the impact of new tools, following their scientific development and validation, can be assessed to allow their introduction and implementation in the Health Service. Italian HTA methods were proposed, and their peculiarities and advantages were discussed for supporting their introduction in the Universal Health Service supply.

The development of high-throughput omics technologies represents an unmissable opportunity for evidence-based prevention (Figure 1). However, the applicability of, and access to, multi-omics tests are still limited in developed countries; these limits must be rapidly overcome. The main obstacles are the rapid increase of knowledge—which is not always open-access (especially for advanced technical competences such as big-data validation and bioinformatics)—and economic investment. Undoubtedly, microbiome research is included in this context. Finally, the significant results and impact generated by the scientific community in a relatively short period of time testify to the realistic and effective prospective of improved omics for disease prevention and general Public Health.

**Figure 1:**
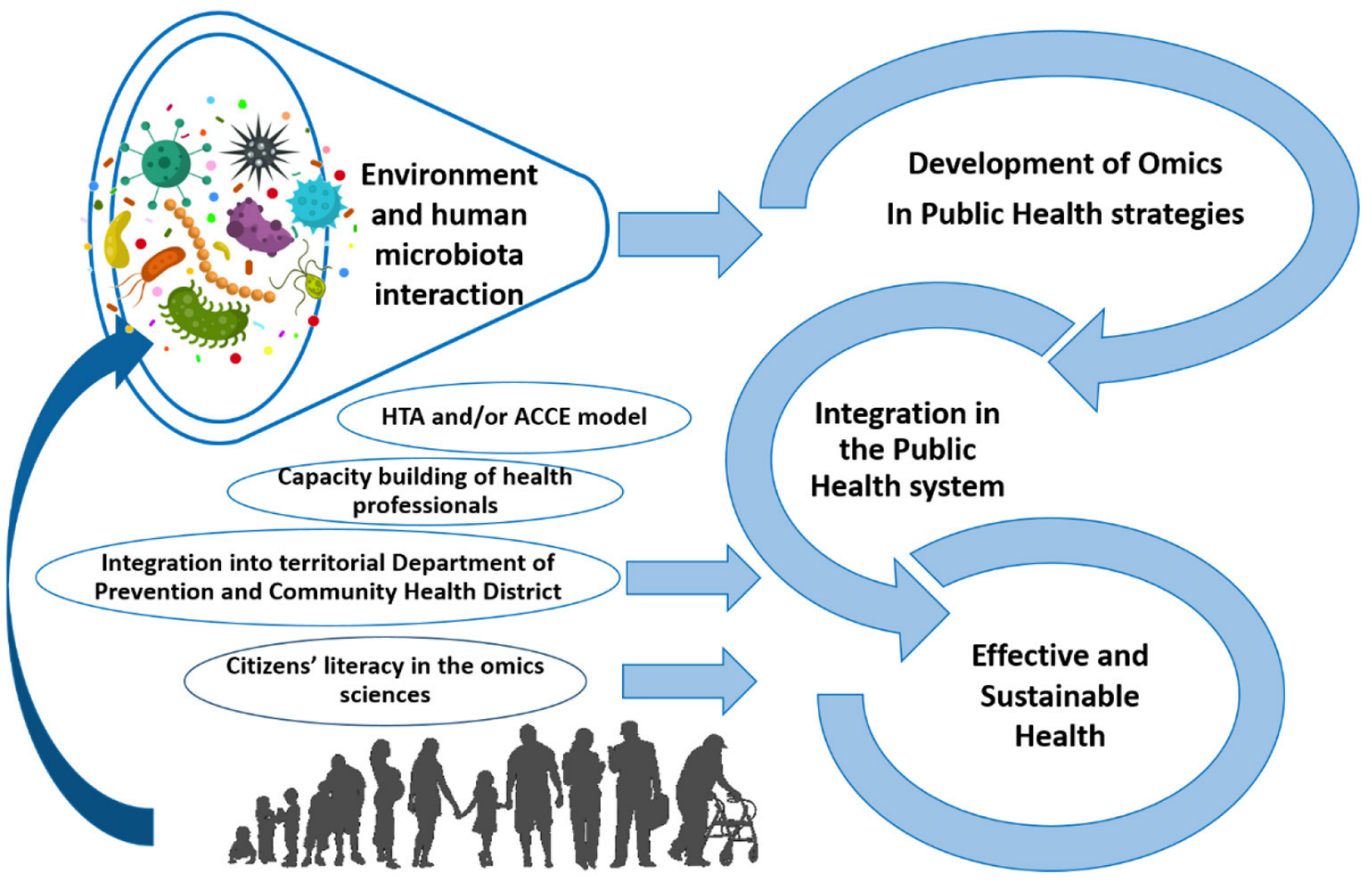
Framework for the development of high-throughput omics technologies, including microbiomics, for evidence-based prevention in public health (taken and modified from Traversi et al., 2021).

## Author contributions

All authors listed have made a substantial, direct, and intellectual contribution to the work and approved it for publication.

## Conflict of interest

The authors declare that the research was conducted in the absence of any commercial or financial relationships that could be construed as a potential conflict of interest.

## Publisher's note

All claims expressed in this article are solely those of the authors and do not necessarily represent those of their affiliated organizations, or those of the publisher, the editors and the reviewers. Any product that may be evaluated in this article, or claim that may be made by its manufacturer, is not guaranteed or endorsed by the publisher.
